# Imaging Characteristics of Dural Arteriovenous Fistulas Involving the Vein of Galen: A Comprehensive Review

**DOI:** 10.7759/cureus.2180

**Published:** 2018-02-11

**Authors:** Mohammad W Kassem, Paul J Choi, Joe Iwanaga, Martin M Mortazavi, R. Shane Tubbs

**Affiliations:** 1 Clinical Anatomy, Seattle Science Foundation; 2 Seattle Science Foundation; 3 California Institute of Neuroscience, Los Robles Hospital and Medical Center; 4 Neurosurgery, Seattle Science Foundation

**Keywords:** vein of galen, aneurysmal malformation, arteriovenous fistula, imaging, diagnosis, prenatal

## Abstract

Vein of Galen aneurysmal malformation (VGAM) is a rare angiopathy, which most commonly presents in infancy. Although very rare, it is associated with high morbidity and mortality rates. In order to minimize such morbid rates, a prompt diagnosis followed by a timely initiation of management is crucial. Multiple antenatal and postnatal imaging techniques for the diagnosis have been described and discussed in the literature. However, to our knowledge, a comprehensive review exploring such a list of imaging options for VGAM has never been established. We aim to review the diagnostic tools to aid in better understanding of the investigative modalities physicians may choose from when treating patients with a VGAM.

## Introduction and background

**Figure 1:**
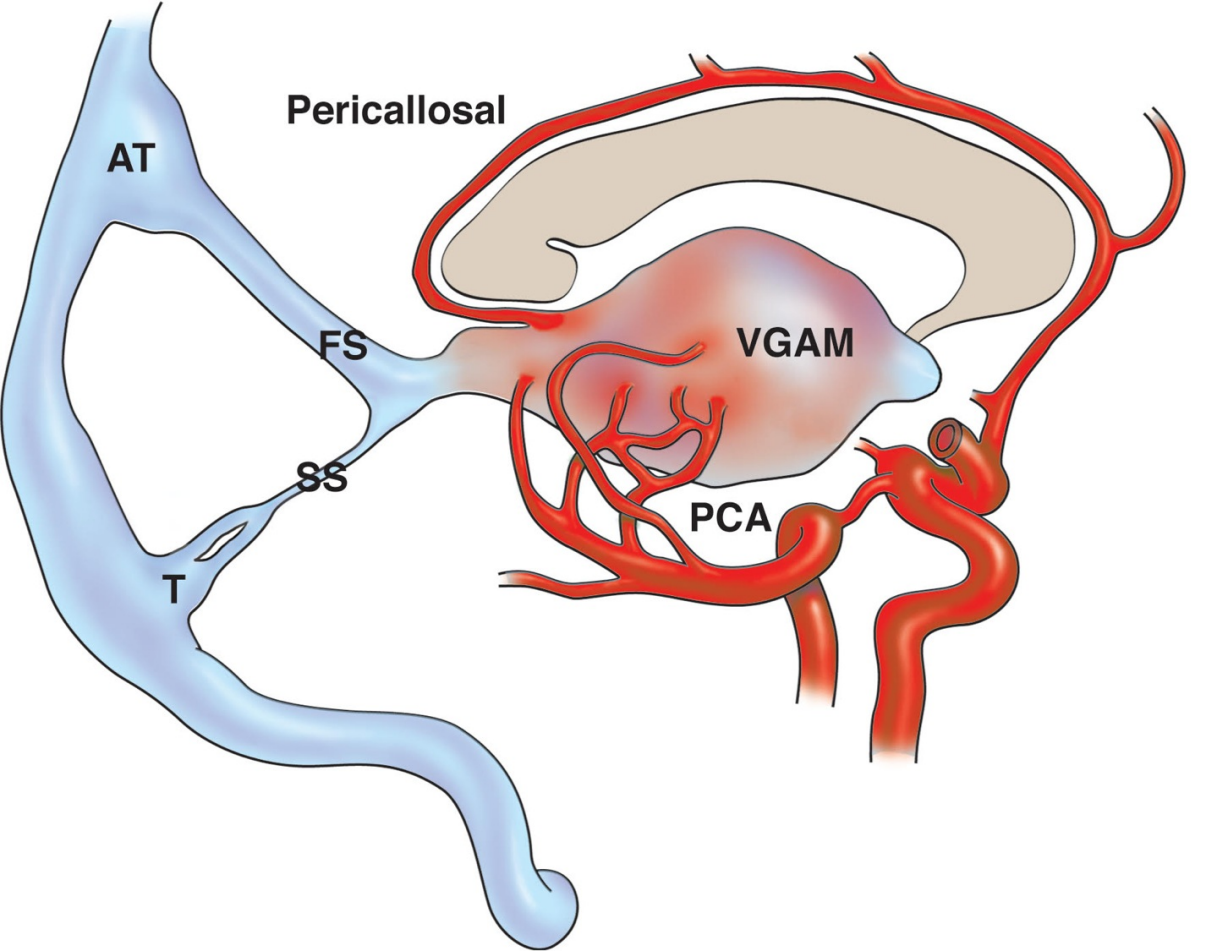
Schematic drawing of the pathomechanism of a vein of Galen aneurysmal malformation Note the pericallosal artery and choroidal branches of the posterior cerebral artery (PCA) feeding the vein of Galen aneurysmal malformation (VGAM). Also note the prominent falcine sinus (FS) and the “accessory torcular” (AT). SS: straight sinus; T: torcular.

Dural arteriovenous fistula (DAVF) is a rare condition that arises from acquired arteriovenous shunts within the dura mater [1-3]. This cerebral arteriovenous malformation involves a single or multiple arterial vessel(s) that feed into a developing choroid, an accumulation of dysplastic vessels, and histologically appears as a congregation of veins, which are arterialized due to the high-pressure arterial system feeding into them. Classically, the choroid can either be fed via a single or by multiple vessel(s) [1, 4]. DAVFs represent 10% to 15% of all cerebrovascular malformations. A rare subtype of a DAVF is the vein of Galen aneurysmal malformation (VGAM), which is also known as the median prosencephalic arteriovenous fistula (Figure 1) [1, 5]. This uncommon subtype of DAVF presents in a more serious and dramatic manner in infancy [1, 6].

VGAMs are more common in males, although Darji et al. claim that they affect boys and girls equally [6]. They make up less than 1% of all intracranial arteriovenous malformations (AVMs) but are 30% of all pediatric intracranial AVMs [7-9]. VGAMs are also the most commonly diagnosed antenatal intracranial AVMs [7]. Their most common presenting symptoms include mild pulsatile tinnitus, hydrocephalus secondary to venous hypertension or aqueduct stenosis, developmental delays, focal neurological deficits, and an intracranial hemorrhage (ICH) [2, 5, 6-7]. Other presentations include epistaxis, proptosis, and less commonly, visual loss from bilateral optic disc atrophy [4].

**Figure 2:**
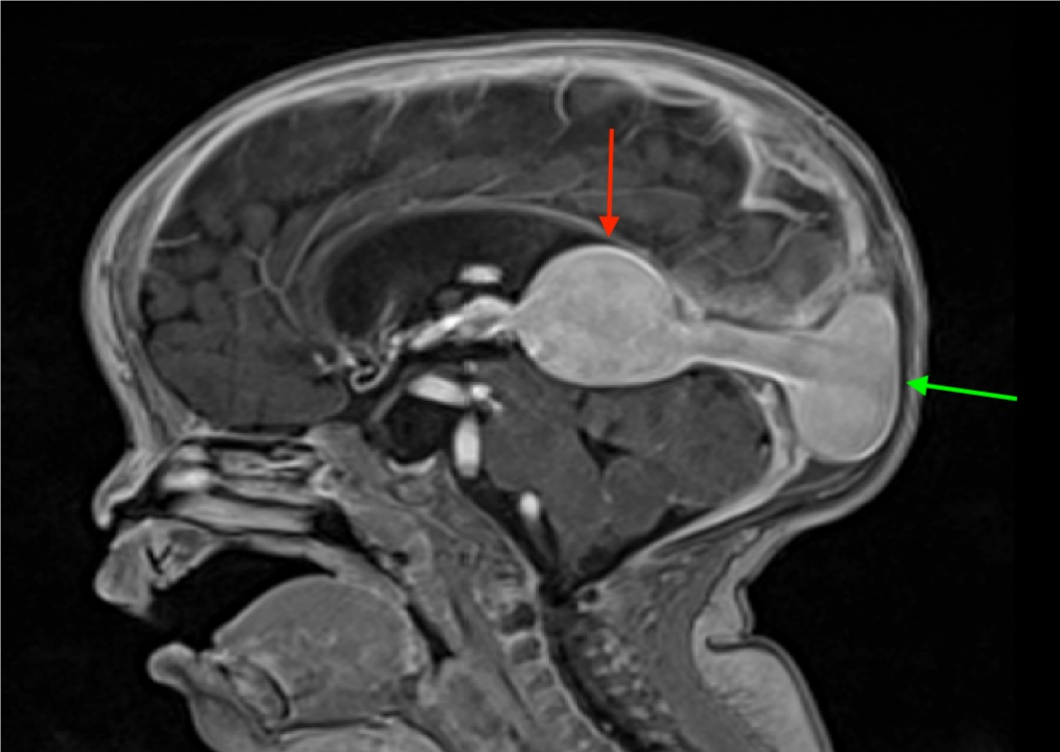
Sagittal T1-weighted MRI of a child with a VGAM (red arrow) Also note the enlarged torcular herophili (TH) (green arrow). MRI: magnetic resonance imaging; VGAM: vein of Galen aneurysmal malformation.

A VGAM (as seen in Figure 2) that presents in the first trimester (6-11 week) of fetal life is of great concern due to its propensity to cause high-output cardiac failure, the result of an increased venous return and subsequent contractility, cardiac output, and eventual high demand for oxygen [4, 6-8, 10]. The patients may have cardiomegaly and pulmonary hypertension, which may lead to fetal hydrops [4, 8, 11]. The VGAM is also known as a rare subtype of falcotentorial DAVF, which is a well-documented cause of an ICH [7, 12]. Identifying VGAM early is crucial in preventing developmental delays, intellectual disabilities, seizures, and a spontaneous ICH [4-5, 7].

**Figure 3:**
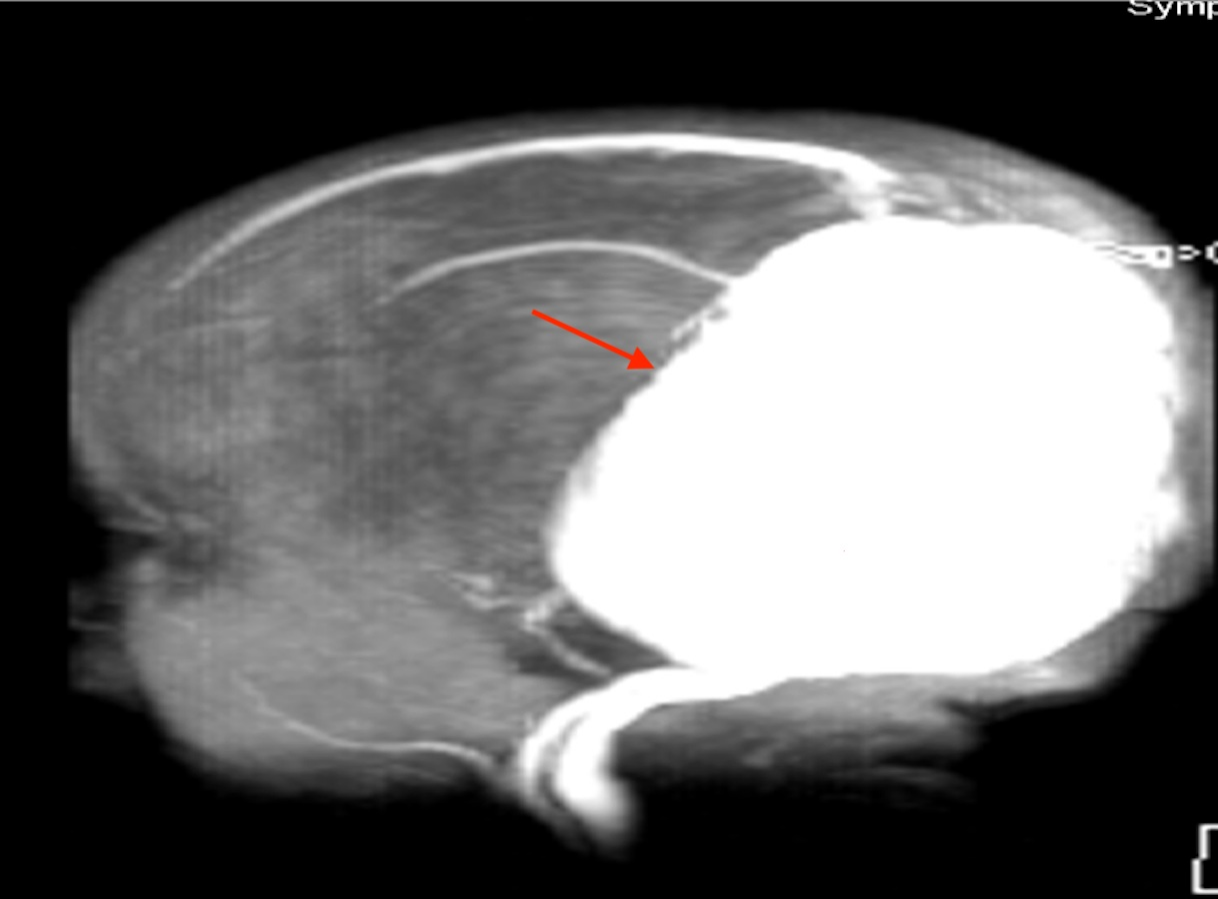
MRI scout image noting the large VGAM (red arrow) seen in the patient shown in Figure 2 MRI: magnetic resonance imaging; VGAM: vein of Galen aneurysmal malformation.

The mortality rate of this condition is as high as 15% if identified timely and managed promptly with endovascular embolization, which is the current treatment of choice for it yields the most favorable outcome [4-5, 9, 13]. However, if such an early detection and an expedited management are not established, a VGAM bestows a dismal 76.7% mortality rate if untreated, and the patients who are treated with a microsurgery take in a mortality rate of 39.4% (Figure 3) [6, 13]. Even if the patient survives, there is a 30% risk of developing a neurological complication [3, 9, 11].

## Review

Imaging modalities for the diagnosis of VGAM

Imaging is a critical aspect in the diagnosis of a VGAM. A VGAM requires a sensitive imaging input for an early detection, which would allow for a careful obstetric monitoring in order to prevent a fatal ICH [1, 5, 11-12, 14]. A prenatal diagnosis of a VGAM offers an opportunity to plan the delivery at a center equipped with a multidisciplinary team who can provide the ideal care [11]. This provides an additional benefit of allowing the parents to prepare, emotionally, for a palliative intervention and avoid unnecessary non-surgical and surgical interventions [15]. Diagnosing a VGAM prenatally can make a significant impact in managing the patient postnatally.

Ultrasound

A two-dimensional real-time ultrasonography can identify an aneurysm as a hypoechoic midline mass posterior to the roof of the third ventricle [2, 6, 8, 15-17]. Using color Doppler technology as an adjunct further demonstrates the hemodynamics, i.e., increased middle cerebral artery (MCA) pressure, high turbulence, and bidirectional blood flow [15], within the aneurysm and aids in ruling out other midline lesions, such as pineal region meningiomas over the vein of Galen [17-19], that appear as a midline solid structure on initial ultrasound [3, 6, 16]. The ultrasonography can also help physicians in identifying other associated anomalies, such as hydrocephalus, ventriculomegaly, and left ventricular dysfunction [5-6, 16-17]. The use of ultrasonography is also of importance in postnatal follow-up on an endovascularly treated VGAM to monitor its potential sequelae [16]. Gun et al. recommend utilization of three-dimensional ultrasonography for antenatal diagnosis of a VGAM for its accuracy in measuring the volume and shape of the lesion [8, 11, 20].

Computed tomography (CT)

Postnatal contrast-enhanced axial CT of the brain can show the detailed multilobulated morphology of the aneurysmal sac, as well as the dilatation of the ventricular system [2-3, 21]. The CT also shows the hypodense periventricular white matter and diffuse cerebral atrophy associated with a VGAM [21]. Cases of spontaneous thrombosis of VGAMs, in which the thrombus sits within the aneurysmal sac, have been reported previously on multiple occasions [2, 21]. Such a thrombus would show up as a heterogeneous mass of hyperdensity, hypodensity, and isodensity, and appears as the “target sign” (peripheral blood flow around the thrombus) under contrast-enhanced CT [21]. Further, the CT technology can highlight dilated collateral veins and hydrocephalus [6, 21]. 

Magnetic resonance imaging (MRI)

**Figure 4:**
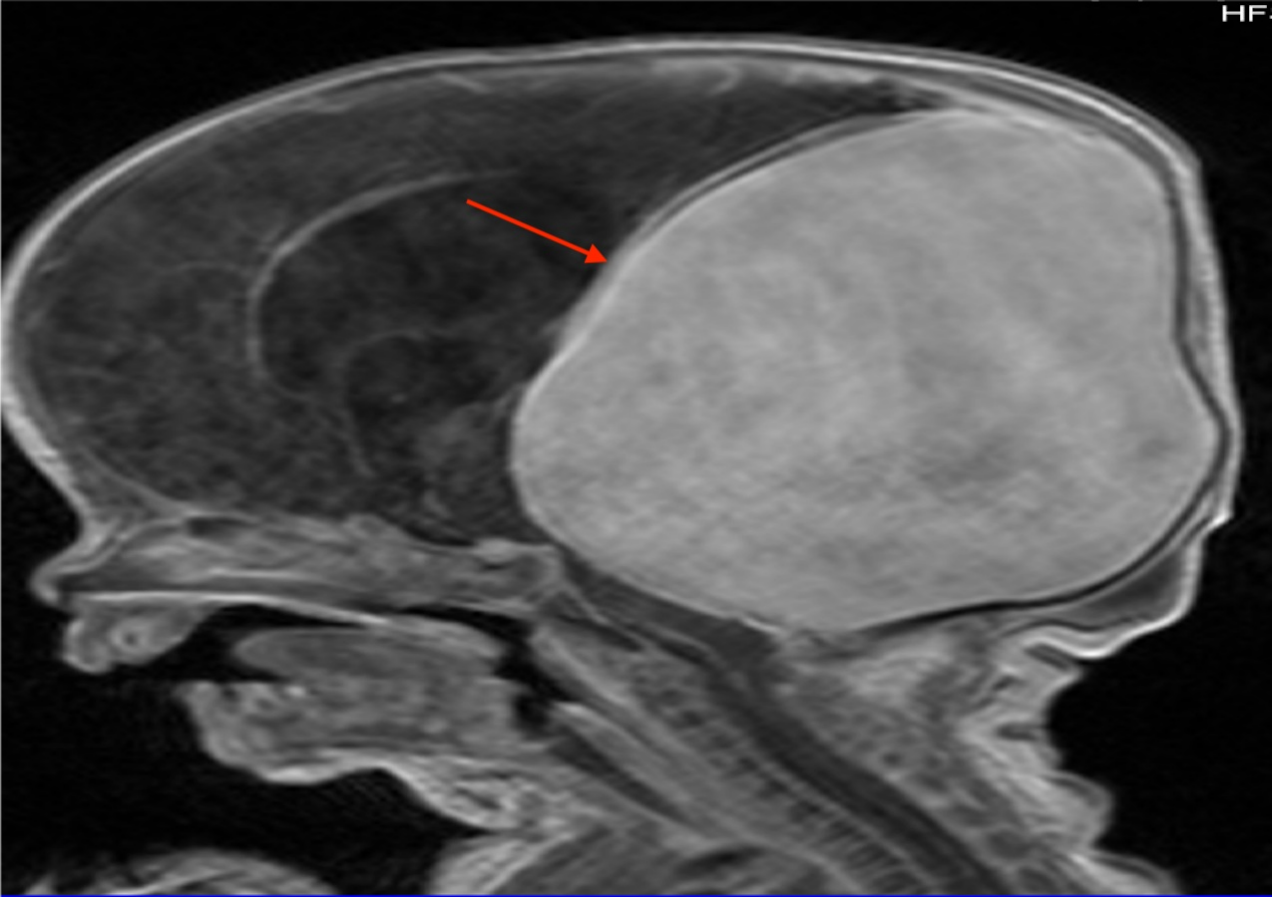
Post-contrasted MRI of a patient with a large VGAM (red arrow) seen in Figures 2 and 3 MRI: magnetic resonance imaging; VGAM: vein of Galen aneurysmal malformation.

**Figure 5:**
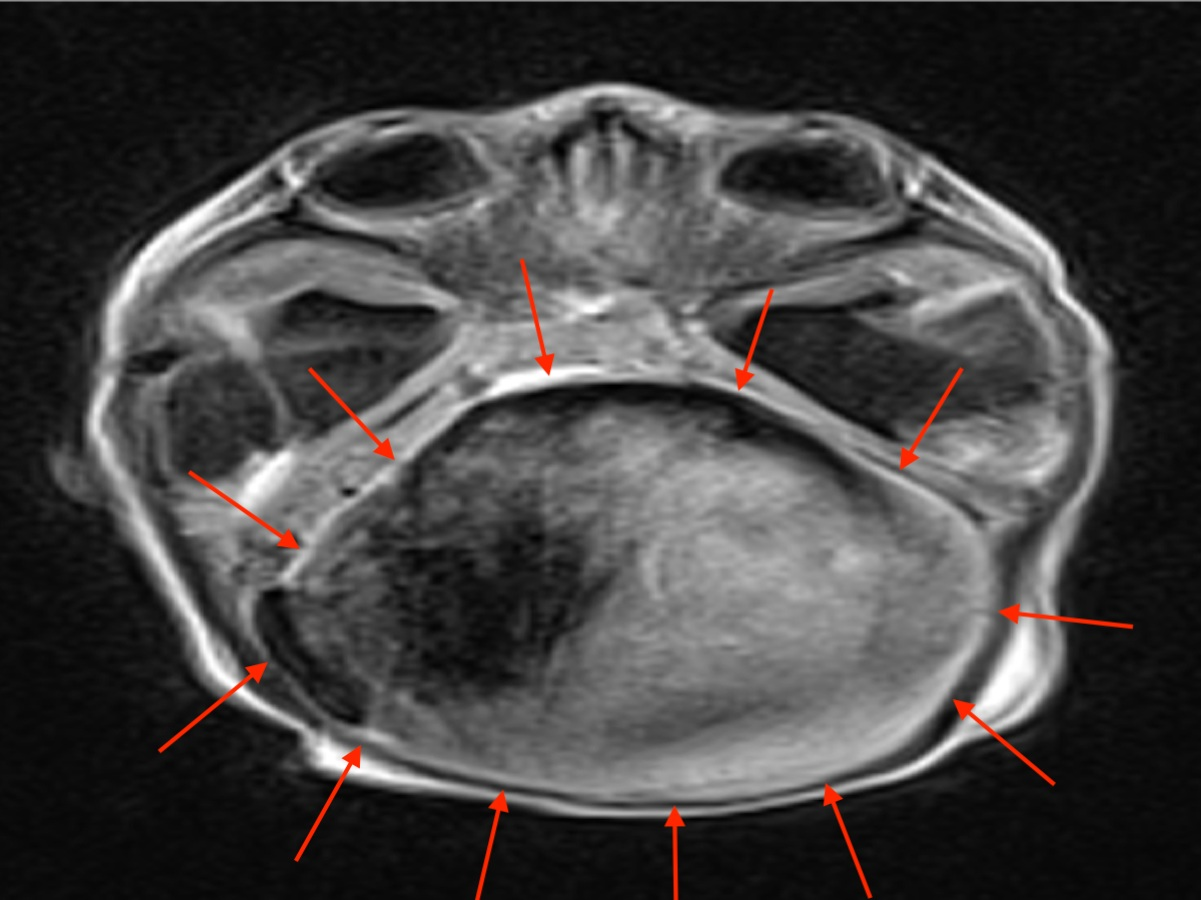
Axial T1-weighted MRI of the patient above with a large VGAM (red arrows) MRI: magnetic resonance imaging; VGAM: vein of Galen aneurysmal malformation.

**Figure 6:**
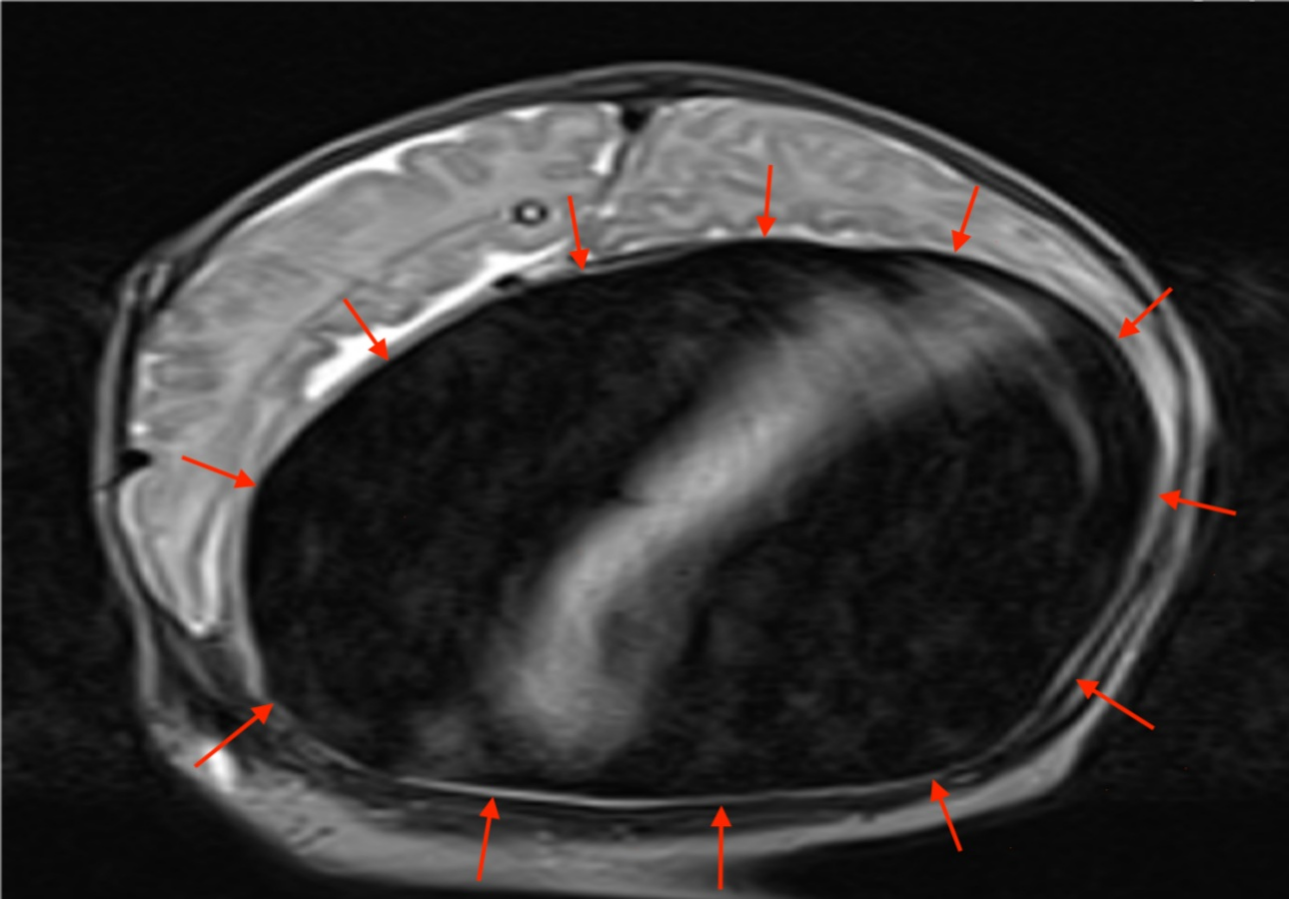
Coronal T2-weighted MRI of the patient above noting the large VGAM (red arrows) MRI: magnetic resonance imaging; VGAM: vein of Galen aneurysmal malformation.

MRI is becoming the imaging modality of choice for VGAM because of its non-invasive nature and its ability to delineate soft tissues effectively i.e., accurately visualizes hydrocephalus, cortical atrophy and injury, and cardiac complications, and to differentiate a VGAM from a cerebral AVM that drains into the vein of Galen (Figures 4-6) [2, 8, 11, 22-23]. Because of its ability to show subtle contrast changes, MRI is ideal for visualizing changes within the cerebral parenchyma and capturing a detailed picture of the ventricular system [5, 22]. MRI is capable of identifying the specific location of a nidus, fistula, venous sac, and the venous vessels [22]. MRI elucidates the major feeding arterial branches more clearly than the traditional CT method [24]. For instance, Saliou et al. reported a fetal MRI brain scan’s accuracy in predicting cardiopulmonary failure at birth and encephalopathic sequelae of VGAM by capturing the M2 and M3 branches of the MCA feeding into the malformation [25].

MRI is also useful in excluding other pathologic lesions such as a choroid plexus cyst and pineal tumor and pinpointing parenchymal injury, which requires an aggressive intervention [8, 15, 17]. In addition, the magnetic resonance angiography (MRA) is a noninvasive alternative imagining choice for the initial evaluation of a suspected VGAM [24]. It has recently been established that fetal MRI is superior to color Doppler in the diagnosis of a VGAM prenatally [8, 23, 26]. Moreover, MRI and MRA have a critical role in understanding the lesion prior and at the time of endovascular embolization respectively [11].

Digital subtraction angiography/fluoroscopy (DSA)

**Figure 7:**
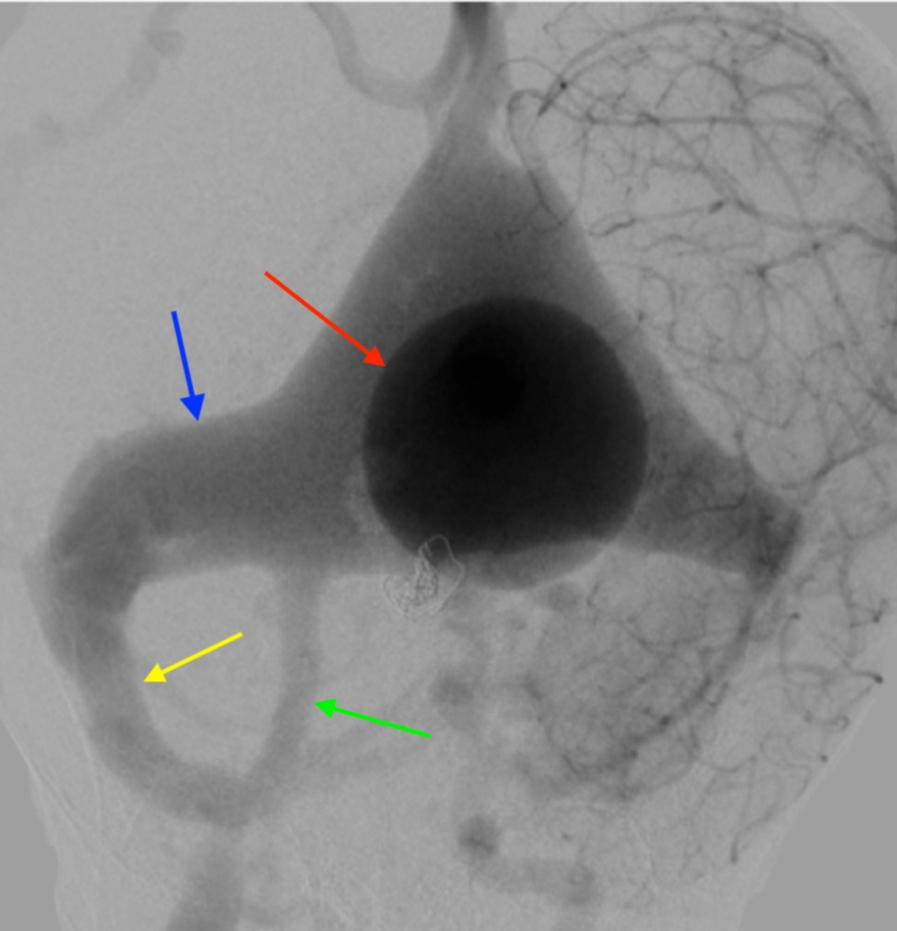
Angiogram, posterior view, noting the VGAM (red arrow), dilated TS (blue arrow), OOS variant (green arrow), and SS (yellow arrow) VGAM: vein of Galen aneurysmal malformation; TS: transverse sinus; OOS: oblique occipital sinus; SS: sigmoid sinus.

**Figure 8:**
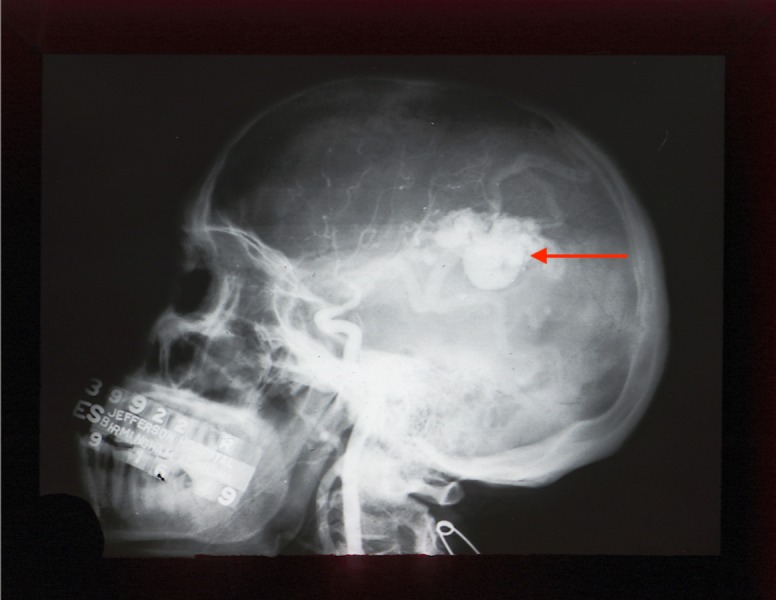
Lateral skull radiograph noting a VGAM (red arrow) VGAM - vein of Galen aneurysmal malformation

DSA is the gold standard in the postnatal diagnosis of a VGAM (Figure 7) [27]. DSA allows for easier visualization of small arteries, which feed into the fistula and it also shows the relationship between the arteriovenous shunt and the venous drainage [6, 27]. The traditional X-ray image of the cranium may aid in the identification of such a condition; it may identify calcification within the wall of the aneurysmal sac and plain radiography of the chest helps rule out congestive heart failure caused by the VGAM (Figure 8) [16, 28].

## Conclusions

VGAM is a rare condition found mainly in the pediatric population and carries a dismal prognosis if undealt promptly with an appropriate imaging study followed by an immediate initiation of an endovascular intervention. Prenatal diagnosis would not only allow time for the transfer of the pregnant mother to a well-equipped medical center for the delivery and care of a neonate with a VGAM, but it may also provide more time for the parents to emotionally prepare for a palliative care and help avoid unnecessary costly interventions. For such reasons, in utero diagnosis is becoming more common with improved fetal imaging technology i.e., three-dimensional ultrasonography and MRI of the fetal brain, which can predict prognosis. An early postnatal diagnosis is also crucial in achieving a better outcome. The appropriate use of advanced imagining techniques allows for an earlier intervention, ultimately leading to improved quality of the children’s remaining decades of life.
